# Technologies and Trends to Improve Table Olive Quality and Safety

**DOI:** 10.3389/fmicb.2018.00617

**Published:** 2018-04-04

**Authors:** Marco Campus, Nurcan Değirmencioğlu, Roberta Comunian

**Affiliations:** ^1^Agris Sardegna, Agricultural Research Agency of Sardinia, Sassari, Italy; ^2^Department of Food Processing, Bandirma Vocational High School, Bandirma Onyedi Eylül University, Bandirma, Turkey

**Keywords:** table olives, novel processing technologies, starter cultures, probiotic strains, non thermal treatments

## Abstract

Table olives are the most widely consumed fermented food in the Mediterranean countries. Peculiar processing technologies are used to process olives, which are aimed at the debittering of the fruits and improvement of their sensory characteristics, ensuring safety of consumption at the same time. Processors demand for novel techniques to improve industrial performances, while consumers' attention for natural and healthy foods has increased in recent years. From field to table, new techniques have been developed to decrease microbial load of potential spoilage microorganisms, improve fermentation kinetics and ensure safety of consumption of the packed products. This review article depicts current technologies and recent advances in the processing technology of table olives. Attention has been paid on pre processing technologies, some of which are still under-researched, expecially physical techniques, such ad ionizing radiations, ultrasounds and electrolyzed water solutions, which are interesting also to ensure pesticide decontamination. The selections and use of starter cultures have been extensively reviewed, particularly the characterization of Lactic Acid Bacteria and Yeasts to fasten and safely drive the fermentation process. The selection and use of probiotic strains to address the request for functional foods has been reported, along with salt reduction strategies to address health concerns, associated with table olives consumption. In this respect, probiotics enriched table olives and strategies to reduce sodium intake are the main topics discussed. New processing technologies and post packaging interventions to extend the shelf life are illustrated, and main findings in modified atmosphere packaging, high pressure processing and biopreservaton applied to table olive, are reported and discussed.

## Introduction

Table olives, which are the most widespread fermented vegetables in the Mediterranean countries, have a great economic significance as a food commodity. Their high nutritional value, the content of bioactive compounds, dietary fibers, fatty acid composition and antioxidants make table olives a valuable functional food. Processes carried out to obtain edible table olives are aimed at the debittering of the fruit, enhancement of the sensory features and to set up chemical-physical and microbiological stability (IOOC, 2004). Agricultural practices carried out in the orchard, including pesticide treatments, soil tillage and harvesting, have an impact on the microbial and chemical quality of the starting material, before processing. From the point of harvesting, a number of pre-processing steps must be carried out to clean the surface of the fruits from soil particles, reduce microbial load, and pesticide residues. Traditionally, olives are washed in tap or chlorinated water, and then passed to processing. Most of alternative are scarcely used in the table olives sector (washing olives with organic acids, ozonated water, etc.), while researchers are studying techniques to improve quality and safety of the material at the pre-fermentation stage (Degirmencioglu et al., 2014; Degirmencioglu, 2016). Different processing technologies give raise to very distinguishable products. While industry demands new methods to improve the processing of table olives, most of innovations are still at the research stage. The most significant advances in table olive processing are represented by the selection of bacteria and yeasts with positive technological traits (Arroyo-López et al., 2012b; Corsetti et al., 2012). In this respect, starter cultures are being presented to the market to enhance fermentation performances. Autochthonous starters are also studied to drive fermentation processes and address consumers demand for typical food with unique and irreproducible characteristics at the same time (Di Cagno et al., 2013; Campus et al., 2015; Martorana et al., 2015). Moreover, table olives are a suitable matrix to convey probiotics (Lavermicocca et al., 2005; De Bellis et al., 2010; Argyri et al., 2016; Rodríguez-Gómez et al., 2017). Innovative, mild non-thermal technologies have been developed in recent years, and some have been studied in table olives to preserve the natural features of the products to a high extent while reducing spoilage and pathogenic microrganisms (Abriouel et al., 2014; Argyri et al., 2014b). The aim of this review was to provide a consolidated view of current technologies and promising advances in the processing of table olives. Starting with the state of the art of technology, the paper will focus on pre-processing technologies, biotechnological innovations, with particular emphasis in the selection and use of microbial starters and probiotics, new processing technologies and processing plants, while the final part will describe innovative post processing preservation techniques.

## Table olive processing technologies up to date

Table olives are processed with the aim of reaching an acceptable level of bitterness, to improve sensory characteristics, and to ensure safety of consumption (IOOC, 2004). Up to date, three main processing technologies are used worldwide, namely: the “Spanish style,” i.e., debittering of the fruits, soaking them in diluted lye solutions, followed by washing steps to remove the excess of lye, and, finally, a partial fermentation in brine, resulting in “treated olives”; the “Californian style,” in which olives are “darkened by oxidation” in lye solutions, alternated with bubbling of air, followed by packaging and retort sterilization; “Natural olives,” in which the olives are put into brine and undergo a spontaneous fermentation, in which yeasts and lactic acid bacteria play a major role (Hurtado et al., 2012). The exact and complete definition of all trade and commercial preparations are described in the “Trade Standard Applying to Table Olives” (IOOC, 2004). Oleuropein is the major phenolic compound in olives, and possesses a strong bitter taste. Oleuropein is degraded to simple phenolic compounds by enzymes (β-glucosidases, esterases), both endogenous and of microbial origin (Garrido-Fernández et al., 1997; Tassou et al., 2002). Moreover, it diffuses from the fruit during lye treatments, washing and soaking in brine for fermentation.

### Treated green olives (spanish style)

Spanish style green olives are obtained from fully developed green fruits collected prior to coloring. Such olives must be firm, sound, resistant to a slight pressure between the fingers, and without marks other than their natural pigmentation. The color of the epicarp may vary from green to straw yellow, according to “Reglamentación Técnico Sanitaria para la elaboración, circulación y venta de las aceitunas de mesa” (Boletín Oficial del Estado BOE, 2001). The main varieties processed according to the Spanish style method are “Manzanilla”, “Hojiblanca” and “Sevillana” (Gordal). After washing and grading, the olives are soaked in a lye solution (usually between 1.3 e 2.6% w/v. NaOH). The lye treatment permeabilizes the outer tissues of the fruit, increasing the diffusion coefficient of hydrophilic compounds (Rodriguez de la Borbolla y Alcala and Rejano, 1979), from olives to solution. The effect is enhanced by increasing concentration and temperature of lye solutions (Fernández-Diez et al., 1985). Bitter compounds such as oleuropein, while diffusing, undergo an alkaline hydrolysis, resulting in simple, non-bitter, phenolic compounds. The traditional process has been subjected to studies focused on the reduction of lye solutions, the shortening of processing times while reducing damages to olives and, as a result, discards. Pretreatment with diluted lye solutions (0.3% NaOH, temperature below 25°C), prior to the main lye treatment in the processing plant, has beneficial effects on the quality of “Manzanilla” olives, especially when olives are harvested and promptly treated (Navarro Rejano et al., 2008), avoiding peeling (detachment of the outer skin from the fruits) and the occurrence of brown spots on the olives surface. Moreover, the treatment eliminates the resting time of “Manzanilla” olives (24–48 h) prior to lye treatment, usually carried out in the traditional method to reduce the occurrence of peeling. The same authors reported that the use of cold (10–14°C), diluted lye solutions allows the prompt treatment of mechanically harvested olives, without further storage. Calcium salts are used both to prevent peeling during the lye treatment and to improve the final texture. During the processing of olives, processing waste with a high pollutant potential (exhausted lye solutions and washing solutions), is produced. Moreover, the operations carried out in Spanish style processing (and in the Californian style) involve a high consumption of water, which is becoming a more and more scarce natural resource. In order to improve the sustainability of lye based processes, different authors have proposed modifications of traditional operations, with the aim of reducing waste waters, chemical and water consumption as well as energy requirements. The current trend is to reuse lye solutions for 5 to 7 cycles (Sánchez Gómez et al., 2006), before discarding. The washing step that follows the lye treatment refers to soaking the olives with tap water. The objective is to dilute the excess of alkali that penetrated into the olives to a minimum level. Washing implies the loss of soluble compounds, such as sugars, which are required in sufficient amount to sustain the fermentation step carried out by Lactic Acid Bacteria (LAB) (Rodriguez de la Borbolla y Alcalá and Rejano, 1978). The current practice has reduced this step to one that takes 12–15 h. Fermentation in brine is the final step of the Spanish style processing. Spontaneous fermentations have been progressively substituted with the use of starter cultures. This topic will be discussed comprehensively in another part of the review.

### Ripe olives processed by alkaline oxidation (californian style)

Californian style processed olives are produced starting with olives harvested at the green-yellow stage. Olives are stored in acidulated solutions in anaerobic conditions (USA) or aired brines added with calcium salts and organic acids (Spain) (Sánchez Gómez et al., 2006). Olives are then oxidized in horizontal stainless steel or plastic and fiber glass containers in which pressurized air is uniformly bubbled (Fernández-Diez et al., 1985) into the solution. Oxidizing treatments are interspersed with treatments with dilute lye solutions, closing the daily cycle of treatments. The aim of the lye treatments (2–7) is the same as the Spanish style. Throughout the operations the olives darken progressively, due to oxidation of ortodiphenols, hydroxytyrosol and caffeic acid (Brenes et al., 1992; García et al., 1992) to their corresponding quinones. The final lye treatment reaches the stone, then olives are washed several times with water to remove the NaOH and lower the pH in the flesh to around 8 (Fernández-Diez et al., 1985). The number of washes can be lowered by adding HCl to the liquid. It is a common practice to inject CO_2_ into the containers to lower the pH to 7.5 before canning (Brenes et al., 1993). To stabilize the dark color, ferrous gluconate is added, at a concentration of 100 ppm of iron in the solution. This phase lasts about 24 h (García et al., 2001). Black olives need a sterilization treatment to ensure microbiological stability. Required lethality at 121°C is F_0_ = 15, *z* = 10. *Clostridium botulinum* is the reference bacteria for thermal treatment of low acid foods, such as oxidized black olives (IOOC, 2004).

### Natural table olives (natural or greek style)

Natural table olives are harvested at the green-yellow stage, or fully ripened, previously washed and graded, then submerged into NaCl solutions (6–10% w/v), on which fermentation takes place, mainly due to the metabolism of autochthonous microbiota present in the olives surface and in the processing plant environment (Romero et al., 2004a). Different variables affect the process, which are both intrinsic, such as the olive cultivar used (Medina et al., 2010), the microbial species present over the fruit surface (Nychas et al., 2002), and technological, particularly brines concentration, processing temperature and disinfection practices (Tassou et al., 2002). Debittering is achieved through the diffusion from fruit to brine of the bitter compound oleuropein, and its enzymatic hydrolysis, carried out by microbial and endogenous enzymes (Garrido-Fernández et al., 1997; Tassou et al., 2002). Spoilage or even pathogenic species could develop during the first stages of the fermentation but they usually rapidly succumb to yeasts and LAB since they are more sensitive to salt concentration and acidification of brines which are determined by metabolic activity exerted mainly by LAB. The growth of LAB depends largely on the processing conditions (Abriouel et al., 2011). Yeasts, depending on the species involved, can exert both positive or negative effects (Arroyo-López et al., 2012a). Fermentations are carried out in plastic vats or fiberglass tanks, scarcely controlling salt concentration, pH and microbial evolution. Products are commercialized through packaging in bags or vats using acidified salt solutions.

## Pre-processing of olives

### Chlorine and Its Alternatives in the surface disinfection of table olive

To ensure microbial safety, table olive processing requires a number of pre-processing steps, such as cleaning, washing, and sanitizing. The objective of these steps are to remove parts of olive tree (stems, leaves, twigs, other debris), soil and pesticide residues, lower the temperature and reduce the undesirable microbial load of the product by thermal, non-thermal, mechanical, or chemical methods. These methods are in common with olives to be processed to obtain olive oil (Ciafardini and Zullo, 2002; Panagou, 2006; Degirmencioglu, 2011a; Degirmencioglu et al., 2011b; Vichi et al., 2015). The surface disinfection methods of olives, prior to processing, can be performed by dipping in or spraying tap water or aqueous antimicrobial solutions (weak organic acids, chlorine and its derivatives, or hydrogen peroxide), by coating with an edible substance, by physical methods, such as ultraviolet light, ultrasounds, electrolyzed water solutions (EWS), ionizing radiations.

#### Chlorine-based agents

Sodium hypochlorite (NaClO), calcium hypochlorite (Ca(ClO)_2_), and chlorine gas (Cl_2_) are extensively used to decrease the initial microbial load of olives surfaces, due to their bactericidal properties (Degirmencioglu et al., 2014; Banach et al., 2015), thus preventing spoilage. In industrial applications, chlorinated water (50–200 ppm as free chlorine, 1–2 min, and pH = 6.0–7.5) are used extensively to wash fruits and vegetables. Studies have shown that chlorine dips can reduce the population of microorganisms below 2 log colony forming unit (cfu), but it has the potential to react with organic materials to form harmful by-products, therefore it should not be used in high doses (Degirmencioglu, 2016).

#### Gaseous chlorine dioxide (ClO_2_)

Chlorine dioxide, up to 3 ppm in high-pH solutions, can reach and penetrate microbial cells better than aqueous sanitizers, and its biocidal effectiveness is due to its oxidation power that is 2.5 times greater than chlorine (Allende et al., 2006; Banach et al., 2015; Food and Drug Administration (FDA), 2016a). However, olives should be rinsed with potable water after the washing step (Ciafardini et al., 1994; Degirmencioglu et al., 2014) because it can cause sensory changes (Banach et al., 2015). Treatment with a ClO_2_ wash (10 ppm) followed by dry salting with 10.0% salt, vacuum packaging, and storage at 4°C was more effective than the use of other washing solutions for controlling LAB counts and suppressing yeasts and molds growth.

#### Weak organic acids

Antimicrobial activity exerted by organic acids is attributed to pH reduction. Acidic pH conditions can alter intracellular metabolic activities of microorganisms. In this regard, the type of acid, food composition, the concentration and temperature of acid solution, and the initial microbial load of food, determine the effectiveness of treatments. In dry salted table olives, acetic and lactic acid solutions (2%) are useful as an alternative to ClO_2_, when salt concentrations in the washing solutions are 5.0 and 10.0% (Degirmencioglu et al., 2014). To exert antimicrobial effect, organic acids, such as acetic or lactic acid, can be used by dipping or spraying techniques, but taking account of a possible negative impact on sensory properties, such as color, flavor, texture, and on nutritional value (Banach et al., 2015). A combination of aqueous sanitizers, such as organic acids, chlorine, chlorine dioxide, sodium chloride (Degirmencioglu et al., 2014), alone or combined with physical treatments, such as ultrasound (Ramos et al., 2013) is advisable to provide the required level of protection (Hurdle technology).

#### Ozone (O_3_)

Ozone is a natural and strong antimicrobial agent with high reactivity and penetrability (Ramos et al., 2013), acting also on deodorization, decolorization, degradation of mycotoxins, and oxidizing pesticides (Ikeura et al., 2011; Tamaki and Ikeura, 2012; Chen et al., 2013; Bajwa and Sandhu, 2014). It is 1.5 times stronger than chlorine (Kiriş and Velioglu, 2016), and, to exhert an effective antimicrobial effect, it is recommended to use it in water containing 0.03–20.0 ppm of ozone, for up to 5 min at a slightly acidic pH (Ramos et al., 2013; Banach et al., 2015). Ozone application has been used as a post-harvest treatment and to regenerate fermentation brines in table olives, determining some microbial reductions and extending the shelf life (Segovia-Bravo et al., 2008; García-García et al., 2014; Tzortzakis and Chrysargyris, 2017). It has also the potential to reduce phenolics content in fermentation brines, responsible for their pollutant potential, lowering concentrations to 15 and 7 mg/L, in acidic (pH 4.0) and alkaline (pH 10.0) conditions, respectively (Segovia-Bravo et al., 2008).

#### Ultraviolet (UV) radiation

UV radiations are classified according to their wavelengths. UV-A ranges from 315 to 400 nm; UV-B, from 280 to 315 nm, and the UV-C, from 100 to 280 nm (Prakash et al., 2000; Artés et al., 2009). UV rays act as an antimicrobial agent against pathogens due to DNA damages, appearing effective in causing cell death (Allende et al., 2006; Birmpa et al., 2013). Nonetheless, at lower doses, microorganisms can remain alive due to their repair mechanisms (Meireles et al., 2016). If the proper precautionary measures are taken, UV is a non-toxic, safe and environment-friendly treatment (Otto et al., 2011), but its use may not be practical due to the presence of organic matter of food or suspended particles in water that could absorb or shield the UV rays (Banach et al., 2015). Therefore, UV-C light (low; 1–4 kJ m^−2^ or high; 10 kJ m^−2^) which has a synergistic effect with other treatments, should be combined with other preservation techniques such as chilling, ozonated water (10 ppm) activated with UV-light, disinfection solution, mild thermal treatments (90 or 15 min at 45°C) and MAP to preserve quality of foods (Allende et al., 2006). Although UV technology has been explored as a post harvest treatment on a wide variety of fruits and vegetables (Ribeiro et al., 2012), very few contributions can be found of its use on table olives. Cedola et al. (2013) used UV as a post processing and post packaging intervention. The UV treatment reduced the total viable count and lactic acid bacteria on Peranzana, but not on Nocellara. On the contrary, the effect on the yeasts was significant for on both olive varieties. UV was used also on packaging material, thus achieving the inactivation of yeasts and molds. Basing on these results, it could be interesting to acquire more scientific data to evaluate UV as a pre-processing technology.

#### Electrolyzed water solutions (EWS)

EWS has a strong bactericidal effect, both at acidic pH (2.1–4.5, oxidation-reduction potential (ORP) higher than 1,000 mV with presence of hypochlorous acid), or at neutral-basic pH (pH; 5.0–8.5, oxidation-reduction potential; 500–700 mV) (Ramos et al., 2013). Gök and Pazir (2011) tested the combination of sanitizing solutions with subsequent UV treatment (without brine) for disinfecting the surfaces of black Gemlik olives. EO treatment (Electrolized Oxydating water, produced by electrolysis of chlorinated water with 15–80 ppm free chlorine) reduced the microbial load up to −1.6 Log cfu/g, at 80 ppm with the product at a distance of 10 cm from the UV lamp. Although EO have a potential to be used to decontaminate olives as an alternative to the conventional ones, some authors have raised limitations to the employment of the technology due to corrosion of equipment, some detrimental effects on the quality of treated food products, and environmental and human health issues (Rahman et al., 2010). These problems are avoided using EWS produced with low chlorine concentrations, with a nearly neutral pH, by electrolysis of a dilute NaCl solution (0.9%), thus producing low concentration electrolyzed water (LcEW) (Rahman et al., 2010).

#### Steam jet-injection

With or without chemical disinfectants (chlorine), steam jet injection is a heat treatment that destroys microorganisms and inactivates enzymes. Although the heat treatment is usually applied for a short time (≈10 s) a decrease in the sensory attributes of foods (Martín-Diana et al., 2007) and a loss or reduction of the bioavailability of some nutrients (Rico et al., 2008) is observed. Studies reported that the heat treatment leads to the partial destruction of the undesirable microbiota of olives surface (10 min at 80°C), inactivates oxidative enzymes (30 min at 90°C), enhances fermentability, supports the development of probiotic cultures, during fermentation (60°C), and increases permeability of olive tissue cells (3 min at 73–74°C) (Argyri et al., 2014a; Grounta et al., 2015; Ramírez et al., 2015b).

#### Ionizing radiations

X-rays, gamma-rays, and electron beams, are capable of producing ions and electrically charged atoms or molecules, acting on water, that forms free radicals, which destroy or inhibit microorganisms (Allende et al., 2006; Ramos et al., 2013). Despite that this method is quite effective in microbial growth control, FDA only approves the use of a maximum level of 1.0 kGy to decontaminate vegetables, to designate them as “fresh” (Ramos et al., 2013). Furthermore, this physical method (0.20–1.00 kGy) has been combined with chemical methods such as chlorinated water (80–200 ppm) and/or by using MAP. These combinations are more efficient than irradiation or chlorination alone, reducing microbial load without adversely affecting sensorial quality of foods (Allende et al., 2006; Ramos et al., 2013; Meireles et al., 2016). Tokuşoğlu (2017) applied gamma irradiation as a post packaging treatment to “Gemlik” olives. The treatment determined a decrease in some nutrients (α-tocopherol, total chlorophyll, and total carotene) during 8 months of shelf life observations.

#### Ultrasounds (US)

Ultrasounds are sonic waves at high amplitude (Otto et al., 2011; Misra, 2015; Meireles et al., 2016) that form cavitation bubbles (Seymour et al., 2002). These bubbles collapse generating the mechanical energy responsible for the disinfecting action (detachment), and the free radicals formation (destruction) (Seymour et al., 2002; Sagong et al., 2011). On the other hand, this treatment is not significantly effective on reducing high level microbial contamination, despite being safe and environmentally friendly (Meireles et al., 2016). Therefore, it should be used in combination with aqueous disinfectants, such as organic acid (Ramos et al., 2013), chlorinated water (Seymour et al., 2002), hot water (55°C) (Allende et al., 2006), which improve the effectiveness of these methods (Seymour et al., 2002; São José et al., 2014). Current literature on olive fruit surface decontamination with US and ionizing irradiations requires more data before their application in table olive technology can be adopted by producers. Remarkably, ultrasound treatment is able to speed up and promote NaOH-free olive debittering, without causing any undesirable changes (Habibi et al., 2015, 2016). Ultrasound is an applicable novel technique minimizing debittering time of olive fruits and decreasing NaOH concentration, thus reducing the number of wash-cycles required for the completion of debittering, and at the same time, resulting in substantial reduction in water usage.

### Removal/reduction of pesticide in table olive

Pesticides are widely used in commercial agriculture to fight weeds, molds, and pests, and increase farm productivity (Kiriş and Velioglu, 2016). Most pesticide residues are retained on the surface of fruits and vegetables peels (Bajwa and Sandhu, 2014; Kiriş and Velioglu, 2016), and as part of Good Agricultural Practice (GAP), pesticides should be implemented at the applicable doses. Attention should be paid to the time between the application of pesticide and harvesting (González-Rodríguez et al., 2011; Bajwa and Sandhu, 2014). Pesticides can be classified as hydrophilic or lipophilic. The latter exhibit higher residual levels in food due to their non-solubility in water (Ikeura et al., 2011; Iizuka and Shimizu, 2014), and most of pesticides residues are retained on fruit and vegetable surface, and may be also absorbed into the flesh (Bajwa and Sandhu, 2014). The various food processing methods such as washing (added with strong oxidizing agents, such as ozone, chlorine dioxide), peeling, brushing, blanching, cooking, boiling, etc., can be used to reduce pesticide contamination (González-Rodríguez et al., 2011; Bajwa and Sandhu, 2014; Misra, 2015; Degirmencioglu, 2016; Qi et al., 2018).

Agrochemicals are used extensively to decrease losses (García-Reyes et al., 2007; Amvrazi and Albanis, 2009; Gómez-Almenar and García-Mesa, 2015). Nevertheless, the misuse of pesticides by farmers can lead to a possible contamination, entailing a risk for the health of consumers and the environment, because olives spontaneously fallen to the ground have higher pesticide levels residues than olives collected directly from the tree (Guardia-Rubio et al., 2006a,b; García-Reyes et al., 2007; Kaushik et al., 2009; Ikeura et al., 2011; Bajwa and Sandhu, 2014; Gómez-Almenar and García-Mesa, 2015).

Results of researches on removal of pesticide residues from olive products documented that pesticides, adsorbed on the dust and adhering to the fruit, may be removed, from no reduction to a 45% decrease, depending on the efficiency of the washing step applied (Cabras et al., 1997; Guardia-Rubio et al., 2006a, 2007a,b,c; García-Reyes et al., 2007). At this stage, the effectiveness of the washing process can be improved by cleaning washing machines every day and changing the washing water with fresh water at the starting of the day (Ruiz-Medina and Llorent-Martínez, 2012), and it is enhanced by using chemical agents (Degirmencioglu, 2016). For that purpose, chlorine based products (10–500 ppm), ozone (1–3 ppm), electrolyzed oxidizing water (EO) alone or with combined chlorine solutions (120 mg/L chlorine, for 15 min), hydrogen peroxide (10–100 ppm), weak and strong acids, potassium permanganate (KMnO_4_, 0.001%), NaCl solution (5–10%), baking soda (NaHCO_3_, 5–10%), vinegar (0.1%) for several minutes, and ultrasonic cleaners (García-Reyes et al., 2007; Kaushik et al., 2009; Bajwa and Sandhu, 2014; Gómez-Almenar and García-Mesa, 2015; Qi et al., 2018) can be effectively used.

Experiences of cleaning technologies applied in the olive oil sector can be translated to the table olive processing. Agrochemicals concentrates in washing water during cleaning, decreasing the washing efficiency over time. The use of FeCl_3_ as coagulant, and activated charcoal (200 mg/L) as adsorbent, is sufficient to eliminate pesticide residues in washing water (Guardia-Rubio et al., 2008).

Chlorine dioxide (ClO_2_) is the most powerful oxidizing agent, and several reports have shown that it can remove or reduce pesticides from fruit and vegetable surfaces. The effectiveness in aqueous solutions depends on its concentration, pH, treatment time and temperature, thickness of olives peel, the initial concentration of pesticide on olives peel, and the types of pesticide (Hwang et al., 2002; García-Reyes et al., 2007; Chen et al., 2014; Kiriş and Velioglu, 2016).

Ozone (O_3_) is a natural substance in the atmosphere and it is generated through a high voltage electrical discharge or by UV light irradiation (Tamaki and Ikeura, 2012). There are several reports on the use of ozone to remove pesticides in foods without causing flavor changes, by immersion in ozonized solutions. It is reported that high pH values, high ozone dosages, combined with ultrasound or UV light are favorable for pesticide degradation (Xiong et al., 2011; Tamaki and Ikeura, 2012; Misra, 2015). However for better removal efficiency, the ozone dosage, treatment time and temperature, bubble size of ozone, and product geometry should be standardized (Wu et al., 2007; Misra, 2015). Kiriş and Velioglu (2016) studied key olive oil processing factors that influence the removal efficiency of pesticide residues with ozone, and they found that ozonated water wash cycles (2 and 5 min) efficienctly removed pesticides, depending on the structural properties of the pesticide itself, olive fruit variety, oil and water content, and oil extraction technology.

The application of intense UV light also can promote the photodegradation of some pesticides by direct photolysis due to their potential to absorb light (Nieto et al., 2009). The authors attempted to develop a simple UV immersion system (200–280 nm, 150 W) to reduce the amount of pesticides in virgin olive oil depending on the treatment time and temperature (15, 20, 25, and 30°C). While these results indicated the possibility of using UV light as an effective, low-cost process for the destruction of pesticides in olive oil and in table olives, no further progress has been reported in this regard.

Ultrasound (US) degradation of pesticides has received much attention in the last years, but the use of US technology for pesticide degradation still remains under-researched. All the studies have indicated the US power (frequency, intensity, amplitude), ambient conditions (temperature, pressure, treatment time), product properties (viscosity and surface tension) and chemical structure of the pesticide, to be the most significant factors influencing the degradation of pesticides. However, long treatment times such as 1-2 h should be performed to achieve significant reduction in pesticide concentration, resulting in significant loss of bioactive compounds and worsening of chemical quality (Misra, 2015).

## Biotechnological innovations

### Starter cultures

Fermentation can occur spontaneously, as a result of the development of the microflora colonizing the environment, the raw material, and the tools with which food is obtained and manipulated, or it can be performed using part of a previous successful fermentation batch to inoculate a new batch (back-slopping), or by addition of starter cultures (Leroy and De Vuyst, 2004). Microorganisms used to obtain fermented foods belong to several taxonomic groups, mostly to LAB, both homo- and heterophermentative, and others such as micrococci, propionibacteria, yeasts, and molds (Bavaro et al., 2017; Bonatsou et al., 2017). It is possible to distinguish selected and natural starter cultures. Selected starter cultures (SSC), which are simpler than natural ones, made up of a low, defined number of species and strains (sometimes they are monocultures), chosen for their strong aptitude for fulfilling the biochemical processes required by each production technology and for their suitability to be grown at a laboratory. SSC are widely applied to different fermented food productions that no longer posses geographic niches and typicity. Since selected strains have strong technological and growth abilities, they become easily dominant over natural microflora when added to raw material in high concentration. The consequent dramatic decrease of microbial biodiversity results in loss of manifold peculiar characteristics and flattening of fermented food sensory profile (Corsetti et al., 2012; Martorana et al., 2015). Industrial level productions are often driven by SSC (Hurtado et al., 2012).

Natural starter cultures (NSC) are made up of microorganisms that colonize production environments, tools and raw materials.

NSC are complex and undefined cultures, consisting of an indefinite number of species and strains, starter and non-starter, coexisting in equilibrium. NCS strains composition is not reproducible in a place other than that of their origin, thus they are thought to be capable of enriching products with peculiar sensory features that bind them to the territory (Leroy and De Vuyst, 2004; Bassi et al., 2015). Usually, NSC characterize the most typical and high quality agri-food products (Martorana et al., 2015).

The main advantages of their use stem primarily from their high biodiversity. Indeed, a large number of strains are capable of following different and complementary metabolic pathways, so that the development of one becomes functional to the development of another, mutually enhancing their abilities (Gatti et al., 2004).

Moreover, biodiversity grants resistance to phages infections on natural cultures, which are usually strain-specific. Indeed, even if one out of the many strains present in the microbial community succumbs to viral attacks, other phage-insensitive individuals will survive and be able to fill the functions that have been carried out by the infected strain until then. On the contrary, fermentations relying on SSC, often made up of one or two strains (Heperkan, 2013), can be impaired by a phage attack, with fatal consequences on the final product (Lanza, 2013).

The main pros and cons of each kind of culture, which should be borne in mind in order to choose the most suitable starter, are summarized in Table 1.

**Table 1:** Pros and cons of selected and natural starter cultures.

**Type of culture**	**Pros**	**Cons**
Selected	Easy to prepare and manage	Poorly flexible system
	Easy check for purity and activity	High sensitivity to phage attack, cultures rotations are needed
	Standardized activity	Not suitable for many PDO, traditional and typical products
Natural	Low sensitivity to phage attack	Problems in the case of predominance of low performances strains
	Better adaptability to the raw material to be processed	Difficult to standardize natural starter culture reproduction, concentrations and performances
	Symbiotic effects	Original balance among species and strains difficult to preserve and check
	Unique sensory quality of the product (the only allowed for many PDO, traditional and typical products)	Difficulties in products sensory quality standardization

Among vegetables, table olives are the most widespread fermented food in Western countries, particularly in the Mediterranean area (Panagou et al., 2008; Wacher et al., 2010; Aponte et al., 2012; Bonatsou et al., 2015, 2017; Tufariello et al., 2015). Although the use of starters is widely diffused in the production of many fermented foods (dairy, meat, sourdough), it is still relatively limited in table olives and in vegetable fermentation, in general (Di Cagno et al., 2008; Aponte et al., 2012; Heperkan, 2013; Iorizzo et al., 2016; Bonatsou et al., 2017). Important table olive varieties, such as Kalamáta, Conservolea, Manzanilla, Sevillana, Hojiblanca, Bella di Cerignola and Ascolana Tenera, are mainly still processed without starter cultures addition (Panagou et al., 2008; Aponte et al., 2010, 2012; Bevilacqua et al., 2010, 2015; Corsetti et al., 2012; De Angelis et al., 2015; Rodríguez-Gómez et al., 2017). However, interest in SSC is increasing in industrial table olives production in Spain, other Mediterranean countries and Argentina (Heperkan, 2013).

Vegetable fermentations are difficult to control, mainly because this kind of raw material cannot be thermal treated without resulting in damage of the product texture, and abnormal phenomena are usually kept under control merely adequately modulating process parameters, such as salt and pH. Starter application, mainly aimed to quicken fruit debittering, contributes to prevent the development of spoilage and pathogenic bacteria, prolonging olive preservation by brine acidification, and improving their sensory properties (Romeo, 2012).

When selecting microorganisms for table olive fermentation, there are many factors to be taken into account. First, the preferability of the species changes depending on the cultivar, type of processing, and geographic area the raw material come from Argyri et al. (2016). Furthermore, microorganism growth rate, ability to survive brine adverse conditions (low temperature, high pH values and NaCl concentration), adhere to olive cuticle, and survive freeze-drying process are criteria to be followed when selecting strains to be used as starter cultures (Heperkan, 2013). Moreover, of no lesser importance, the resistance to organic acids and polyphenols, ability of rapidly metabolizing fermentable substrates and oleuropein, aptitude to produce aromas, synthesize bacteriocins, and colonize the brine by countering the development of undesired microorganisms (i.e., *Enterobacteriaceae, Clostridium, Pseudomonas, Staphylococcus*, and *Listeria*), must be considered (Bevilacqua et al., 2010; Corsetti et al., 2012; Bleve et al., 2015).

Besides the technological efficency of microorganisms, as for all the substances intentionally added to foods, their safety must be checked and proven, unless they are recognized among qualified experts, as having been adequately shown to be safe under the conditions of their intended use (Generally Recognized As Safe, GRAS) (Food and Drug Administration (FDA), 2016b), or have the QPS (Qualified Presumption of Safety) status (European Food Safety Authority [EFSA], 2017).

In literature dealing with biodiversity of table olive microflora, it is possible to find exhaustive lists of bacteria, yeasts and molds species, identified both with classical and molecular, culture-dependent and –independent, techniques (Bautista-Gallego et al., 2011b, 2013a; Botta and Cocolin, 2012; Corsetti et al., 2012; Heperkan, 2013; Campus et al., 2015; De Angelis et al., 2015; Bavaro et al., 2017; Bonatsou et al., 2017; Comunian et al., 2017).

Table olive starter cultures, so far mainly developed for Spanish-style processing, are made up of one or two strains belonging to facultative heterofermentative mesophilic lactobacilli species, above all *Lactobacillus plantarum, Lactobacillus pentosus*, less frequently, *Lactobacillus paraplantarum, Lactobacillus casei, Lactobacillus brevis, Leuconostoc mesenteroides, Pediococcus acidilactici* and, more rarely, Enterococci. In some cases, bacteria have been associated with yeasts, such as *Debaryomyces spp., Saccharomyces spp., Candida spp., Pichia spp*., *Rhodotorula spp*., in order to favor and enhance their development (De Castro et al., 2002; Corsetti et al., 2012; Heperkan, 2013; Argyri et al., 2016; Bonatsou et al., 2017.

### Autochthonous LAB starter cultures

As stated above, the use of starters in table olive fermentation is quite recent and not common yet. Back-slopping, a technological expedient borrowed from other fermented food technologies (such as dairy products, sourdough and wine), relying on autochthonous microflora, has been the first attempt to improve and speed up table olive fermentation process, and can be considered a precursor of the starter culture method (Aponte et al., 2012; Corsetti et al., 2012). However, this practice is not risk-free. Indeed, together with useful autochthonous microflora, even pathogen or spoilage bacteria could be potentially inoculated, and allowed to reach high and dangerous concentrations in the new food batch if the fermentation is unsuccessful. This is why back-slopping is being progressively abandoned in various fermentation processes and, in some cases, it has become even illegal (New Zealand Food Safety Authority [NZFSA], 2008).

In recent years, the consumer demand for more traditional and homemade products, with unique characteristics, different and distinguishable from the others present on the market, is increasing (Corsetti et al., 2012; Medina et al., 2016). To satisfy this demand, overcome the back-slopping drawbacks and avoid the standardization of the product as a result of using commercial SSC, it has been thought to isolate autochthonous strains from successful natural fermentations, test them for their technological abilities, and use the most promising strains as starters. Indeed, autochthonous strains are generally considered to be more adapted to the microflora naturally present in the raw material to be processed than allochthonous ones, and therefore they are able to dominate the microbiota, counteract spoilage microflora, and drive the fermentation from the very early phases of the process (Di Cagno et al., 2008; Aponte et al., 2012; Bevilacqua et al., 2015). Furthermore, autochthonous starters cultures (ASC) are the most suitable for PDO, IGP productions (often the only starters allowed by PDO specifications), as they are considered to be able to bind the product to the region where it comes from. Notwithstanding this, ASC are still less widespread than commercial SSC, particularly in table olive production, and there are only a few studies dealing with autochthonous starter selection (Leal-Sánchez et al., 2003; Di Cagno et al., 2008, 2013; Aponte et al., 2012; Campus et al., 2015; Tataridou and Kotzekidou, 2015; Tufariello et al., 2015). However, it should be kept in mind that the use of 1–3 selected strains, though autochthonous, is not a guarantee of good fermentation performances and counter-action of spoilage/pathogenic microflora, nor results in peculiar qualities that make the product clearly distinguishable from other analogous ones produced in different geographical areas (Aponte et al., 2012; Durante et al., 2017). As reported above, NSC, because of the complexity of strains that make them up, with different technological abilities and metabolic pathways, are considered as the most suitable starter to grant a specific identity to a fermented food (Leroy and De Vuyst, 2004; Bassi et al., 2015). Martorana et al. (2015) recently reported that ASC can be effectively used to drive the fermentation process of Nocellara del Belice olives, when used as a “Pied de cuve,” both strengthened or not with the inoculum of authoctonous *L. pentosus* strain.

Therefore, the back-slopping strategy can be a good starting point to be enhanced and implemented following a new technological approach, in order to keep its advantages and avoid the drawbacks. A compromise between back-slopping and a SSC (both allochtonous and autochthonous) might be a “semi-natural” ASC consisting of an undefined number of strains. The Selected Inoculum Enrichment (SIE) method, recently proposed and applied to *Tonda di Cagliari* table olives (a cultivar of Sardinia island, Italy) represents a new concept in the use of LAB starters in table olive processing (Campus et al., 2015, 2017; Comunian et al., 2017). Indeed, with the SIE method the number of desirable microorganisms initially present in the brined olives was increased, ensuring a more reliable and faster process than spontaneous fermentation, inoculating only useful bacteria. This complex culture was added to the natural microflora of a new fermentation process without operating any previous selection of particular strains.

Such a mix of autochthonous strains have undergone, as a whole, a natural selection that have made them more adapted to the specific brine conditions. It was observed to be able to counteract spoilage microflora and preserve the product better than allochthonous SSC and natural microbiota, colonizing the environment from the very early stage of fermentation until its end (Campus et al., 2015; Comunian et al., 2017). Therefore, SIE could be a successful technology to be applied at an industrial scale to obtain fermented products, not only table olives, as good as the spontaneously fermented ones, while still guaranteeing safety, quality constancy and reproducibility.

### Yeasts with positive technological traits

Yeasts are part of table olive natural microbiota, coexisting with LAB during the whole fermentation process, but with a wider species biodiversity.

The yeast species detected in table olive fermentation, their prevalence and dynamics change depending on the geographic area, cultivar, interaction with other microorganisms (LAB) and processing technology (salt concentration, temperature, pH, nutrients, oxygen) (Bleve et al., 2014; Leventdurur et al., 2016). In general, higher salt concentrations and phenolic compounds or low pH levels favor yeast development to the detriment of LAB (Bautista-Gallego et al., 2015; Benítez-Cabello et al., 2015; Porru et al., 2018). Therefore, their involvement is particularly important in natural olives, when fruits are not lye treated and phenolic compounds partly inhibit LAB development (Arroyo-López et al., 2012b). Moreover, the presence of the various yeasts species was observed to vary even during the whole fermentation time (their number increased during the fermentation), because of and depending on their abilities to adapt to the evolution of the physico-chemical conditions of olives and brines (Pereira et al., 2015; Arroyo-López et al., 2016).

A list of the yeast species most frequently reported in recent literature (from 2006 up today), as part of the microbiota colonizing 20 table olives cultivars, processed in 6 countries (Spain, Portugal, Greece, Italy, Tunisia, and Turkey), is reported in Table 2.

**Table 2:** List of the most frequently cited yeasts species in recent literature.

**Species**	**Country and cultivar**						**References**
	**Greece**	**Italy**	**Portugal**	**Spain**	**Tunisia**	**Turkey**	
*Aureobasidum pullulans*	Conservolea		Manzanilla				Nisiotou et al., 2010; Alves et al., 2012
*Bullera variabilis*					Greek style black olives		Fendri et al., 2013
*Candida cf apicola*				Aloreña			Abriouel et al., 2011
*Candida atlantica*						Gemlik	Leventdurur et al., 2016
*Candida boidinii*	Conservolea	Bosana, Nocellara del Belice, Leccino, Istrana nera, Peranzana, Cellina di Nardò	Negrinha de Freixo	Arbequina, Aloreña		Gemlik	Arroyo-López et al., 2006; Hurtado et al., 2008; Nisiotou et al., 2010; Tofalo et al., 2013; Bleve et al., 2014; Martorana et al., 2015; Pereira et al., 2015; Leventdurur et al., 2016; Porru et al., 2018
*Candida butyri/aaseri*	Conservolea		Manzanilla	Spanish style green olives, Arbequina		Gemlik	Hurtado et al., 2008; Nisiotou et al., 2010; Alves et al., 2012; Lucena-Padrós et al., 2014; Leventdurur et al., 2016
*Candida diddensiae*		Bosana, Nocellara del Bellice	Manzanilla	Arbequina, Aloreña			(Arroyo-López et al., 2006; Hurtado et al., 2008; Alves et al., 2012; Martorana et al., 2015; Porru et al., 2018)
*Candida glabrata*				Manzanilla			Hernández et al., 2007
*Candida holmii*				Aloreña			Arroyo-López et al., 2006
*Candida humicola*				Manzanilla			Hernández et al., 2007
*Candida inconspicua*				Manzanilla			Hernández et al., 2007
*Candida ishiwadae*		Cellina di Nardò					Tofalo et al., 2013
*Candida maris*				Manzanilla			Hernández et al., 2007
*Candida membranifaciens*		Nocellara del Belice		Arbequina			Hurtado et al., 2008; Martorana et al., 2015
*Candida norvegica*			Negrinha de Freixo				Pereira et al., 2015
*Candida oleophila*			Manzanilla				Alves et al., 2012
*Candida parapsilosis*		Brandofino, Manzanilla, Nocellara del Belice, Passanulara		Arbequina; Manzanilla			Hernández et al., 2007; Hurtado et al., 2008; Aponte et al., 2010
*Candida rugosa*				Manzanilla			Hernández et al., 2007
*Candida silvae*	Conservolea						Nisiotou et al., 2010
*Candida sorbosa*				Manzanilla			Arroyo-López et al., 2012a
*Candida sp*.	Halkidiki						Grounta et al., 2015
*Candida tartarivorans*		Cellina di Nardò					Bleve et al., 2014
*Candida thaimueangensis*				Spanish style green olives			Lucena-Padrós et al., 2014
*Candida tropicalis*			Negrinha de Freixo				Pereira et al., 2015
*Candida zeylanoides*				Manzanilla			Hernández et al., 2007
*Cryptococcus laurentii*				Manzanilla	Greek style black olives		Hernández et al., 2007; Fendri et al., 2013
*Cryptococcus albidus*					Greek style black olives		Fendri et al., 2013
*Cystofilobasidium capitatum*	Conservolea						Nisiotou et al., 2010
*Cyteromyces matritensis*			Manzanilla				Alves et al., 2012
*Debaryomyces carsonii*		Cellina di Nardò					Bleve et al., 2014
*Debaryomyces etchellsii*		Leccino					Bleve et al., 2014
*Debaryomyces hansenii*	Conservolea, Halkidiki, Kalamàta	Cellina di Nardò	Negrinha de Freixo	Manzanilla	Greek style black olives		Hernández et al., 2007; Nisiotou et al., 2010; Fendri et al., 2013; Bleve et al., 2014, 2015; Grounta et al., 2015; Pereira et al., 2015
*Dekkera bruxellensis*				Aloreña	Greek style black olives		Hernández et al., 2007; Fendri et al., 2013
*Galactomyces reessii*			Negrinha de Freixo				Pereira et al., 2015
*Geotrichum candidum*				Manzanilla; Aloreña			Arroyo-López et al., 2006, 2012a
*Hanseniaspora guilliermondii*				Aloreña			Arroyo-López et al., 2006
*Issatchenkia occidentalis*				Aloreña			Arroyo-López et al., 2006
*Kluyveromyces lactis*				Arbequina			Hurtado et al., 2008
*Kluyveromyces marxianus*				Manzanilla			Hernández et al., 2007
*Metschnikowia pulcherrima*	Conservolea						Nisiotou et al., 2010
*Meyerozyma sp*.						Gemlik	Leventdurur et al., 2016
*Nakazawaea molendini-olei*		Bosana					Porru et al., 2018
*Pichia anomala* (current name *Wickerhamomyces anomalus*)	Conservolea, Kalamàta	Bosana, Nocellara del Belice, Cellina di Nardò, Bella di Cerignola		Arbequina, Manzanilla	Gemlik		Hernández et al., 2007; Hurtado et al., 2008; Nisiotou et al., 2010; Tofalo et al., 2013; Bleve et al., 2014, 2015; Grounta et al., 2015; Martorana et al., 2015; Leventdurur et al., 2016; Porru et al., 2018
*Pichia galeiformis*		Peranzana, Nocellara del Belice, Cellina di Nardò		Gordal, Manzanilla			Arroyo-López et al., 2012a; Tofalo et al., 2013; Benítez-Cabello et al., 2015
*Pichia guilliermondii*	Halkidiki, Conservolea	Castriciana, Manzanilla, Passanulara	Negrinha de Freixo	Manzanilla			Hernández et al., 2007; Aponte et al., 2010; Nisiotou et al., 2010; Grounta et al., 2015; Pereira et al., 2015
*Pichia kluyveri*	Conservolea	Brandofino, Castriciana, Manzanilla, Nocellara del Belice, Passanulara		Arbequina			Hurtado et al., 2008; Aponte et al., 2010; Nisiotou et al., 2010
*Pichia kudriavzevii*					Greek style black olives	Gemlik	Leventdurur et al., 2016
*Pichia farinosa*							Fendri et al., 2013
*Pichia manshurica*	Conservolea	Peranzana, Nocellara del Belice, Cellina di Nardò	Negrinha de Freixo				Nisiotou et al., 2010; Tofalo et al., 2013; Pereira et al., 2015
*Pichia membranifaciens*	Conservolea, Kalamàta	Cellina di Nardò e Leccino	Negrinha de Freixo	Gordal, Arbequina	Greek style black olives		Hurtado et al., 2008; Nisiotou et al., 2010; Fendri et al., 2013; Bleve et al., 2014, 2015; Benítez-Cabello et al., 2015; Pereira et al., 2015
*Pichia minuta*				Arbequina			Hurtado et al., 2008
*Pichia rhodanensis*				Arbequina			Hurtado et al., 2008
*Rhodotorula diobovatum*	Conservolea						Nisiotou et al., 2010
*Rhodotorula glutinis*			Negrinha de Freixo	Arbequina, Manzanilla, Aloreña			Arroyo-López et al., 2006; Hernández et al., 2007; Hurtado et al., 2008; Pereira et al., 2015
*Rhodotorula graminis*			Negrinha de Freixo	Aloreña			Arroyo-López et al., 2006; Pereira et al., 2015
*Rhodotorula minuta*				Manzanilla			Hernández et al., 2007
*Rhodotorula mucilaginosa*	Conservolea		Manzanilla	Spanish style green olives			Nisiotou et al., 2010; Alves et al., 2012; Lucena-Padrós et al., 2014
*Saccharomyces cerevisiae*	Kalamàta, Conservolea	Bosana, Cellina di Nardò, Leccino, Istrana nera/bianca, Peranzana, Nocellara del Belice, Bella di Cerignola	Negrinha de Freixo, Manzanilla	Spanish style green olives, Aloreña, Manzanilla			Arroyo-López et al., 2006; Hernández et al., 2007; Nisiotou et al., 2010; Abriouel et al., 2011; Alves et al., 2012; Tofalo et al., 2013; Bleve et al., 2014, 2015; Lucena-Padrós et al., 2014; Pereira et al., 2015; Porru et al., 2018
*Saccharomyces sp*.						Gemlik	Leventdurur et al., 2016
*Saturnispora mendoncae*				Spanish style green olives			Lucena-Padrós et al., 2014
*Schwanniomyces etchellsii*						Gemlik	Leventdurur et al., 2016
*Sporobolomyces roseus*					Greek style black olives		Fendri et al., 2013
*Torulaspora delbruekii*				Manzanilla	Greek style black olives		Hernández et al., 2007; Fendri et al., 2013
*Trichosporum cutaneum*				Manzanilla			Hernández et al., 2007
*Zygoascus hellenicus*						Gemlik	Leventdurur et al., 2016
*Zygosaccharomyces bailii*				Aloreña	Greek style black olives		Arroyo-López et al., 2006; Fendri et al., 2013
*Zygosaccharomyces mrakii*		Leccino	Manzanilla				Alves et al., 2012; Bleve et al., 2014
*Zygotorulaspora mrakii*		Bosana					Porru et al., 2018
*Zygowilliopsis californica*	Conservolea						Nisiotou et al., 2010

*Candida boidinii, Debaryomyces hansenii*, and *Pichia membranifaciens* resulted the most geographically diffused species, since they were detected in all the countries, but one (*D. hansenii* was not detected in Turkey, while *C. boidinii* and *P. membranifaciens* were not in Tunisia). Among the other species reported in Table 2, *Candida, Pichia* (included *Wickerhamomyces anomalus*, current name of *Pichia anomala)*, and *Saccharomyces* were the most frequent genera detected. The other genera (apart from *Rhodotorula*, which was detected in 4 cultivars from Greece, Portugal and Spain), were found only in 1–2 cultivars.

The presence of yeasts can have positive or negative consequences on table olive sensory characteristics and shelf-life; (Nisiotou et al., 2010; Arroyo-López et al., 2012b,c). Indeed, though they can be responsible of clouding of brines, off-flavors, off-odors, and gas-pocket defects. For instance, among frequently isolated species strains of *S. cerevisiae* and *W. anomalus* have been thought to be responsible for producing high amounts of CO_2_, causing gas-pocket defects, while *D. hansenii* and some species of *Rhodotorula* genus showed the ability to degrade fruits cell wall polysaccharides or to produce polygalacturonases causing olives softening (Arroyo-López et al., 2008).

However, strains belonging to the same yeast species can significantly contribute to the improvement of table olive quality and safety. Indeed, they have a role in: (a) favoring useful bacteria growth, thus enhancing lactic acid production needed to preserve the product from spoilage microorganisms; (b) acting as biocontrol agents against fungi and other undesirable yeast and bacteria species; (c) producing compounds that positively affect flavor and texture (i.e., ethanol, glycerol, esters, other volatile compounds, free fatty acids); (d) biodegrading polyphenols (such as oleuropein which gives olives a bitter taste); (e) preventing unsaturated fatty acid oxidation and peroxide formation (catalase positive yeast strains) (Arroyo-López et al., 2008, 2012b; Bevilacqua et al., 2013; Bleve et al., 2014).

In recent years, the interest in studying and developing yeast starter cultures for table olive processing, both alone or in combination with LAB is growing (Psani and Kotzekidou, 2006; Arroyo-López et al., 2008, 2012b; Nisiotou et al., 2010; Bevilacqua et al., 2013, 2015). Even a sequential inoculation strategy (firsly yeasts, then bacteria) was developed to take advantage of the beneficial effect exerted by yeast on LAB growth (Tufariello et al., 2015). Furthermore, yeasts ability to favor mixed species (bacteria and yeast) biofilm formation, both on biotic (drupes) and abiotic (fermenter vats) surfaces, was extensively studied in table olives processing. *C. boidinii, C. sorbosa, D. hansenii, Geotrichum candidum, Pichia galeiformis, P. guilliermondii* and *W. anomalus* were the yeast species proved to be the most effective ones in favoring mixed species biofilm formation (particularly on olive skins) especially in combination with *Lb. pentosus*. Indeed, they displayed capability to develop a propitious environment assuring *Lb. pentosus* survival, enhancing its development and starter activity (Arroyo-López et al., 2012a; Domínguez-Manzano et al., 2012; Grounta and Panagou, 2014; Benítez-Cabello et al., 2015; Grounta et al., 2015; León-Romero et al., 2016; Porru et al., 2018). LAB and yeast populations of the biofilms adhering to the olives skin were estimated in about 6–7 and 3–5 log cfu/g, respectively (Grounta and Panagou, 2014; Benítez-Cabello et al., 2015), while those colonizing olive fermentation vessels were quantified in 3.0–4.5 and 4.0–4.6 log cfu/cm^2^ (LAB and yeast counts, respectively). Biofilm populations, particularly yeasts, were able to survive cleaning treatments of vessels and were still alive even when vessels were left to dry for 60 days (between 2.76 and 3.94 log cfu/cm^2^, while LAB were not detectable; Grounta et al., 2015).

Furthermore, it is noteworthy that the genera/species most frequently isolated from not inoculated brines and drupes are also the ones that showed many of the positive technological traits that make yeast strains good candidates as starter culture for table olives. Indeed, *C. boidinii* which belongs to the most widespread genuswas recognized to have a positive effect on olive flavor, by means of the formation of esters from drupes free fatty acids thanks to its strong lipase and esterase activity (Hernández et al., 2007; Rodríguez-Gómez et al., 2010; Bautista-Gallego et al., 2011b; Arroyo-López et al., 2012c; Pereira et al., 2015). While, *S. cerevisiae* and several species of the *Pichia* genus (catalase positive yeasts) showed antioxidant activity, useful to protect fruits from unsaturated fatty acid oxidation and peroxide formation (Hernández et al., 2007). Strains of *W. anomalus*, a species with a strong tendency to grow in harsh environmental conditions, such as low pH and high NaCl concentrations, in addition to high lipase and esterase activity, showed strong β-glucosidase activity (Bautista-Gallego et al., 2011b; Arroyo-López et al., 2012c; Romero-Gil et al., 2013; Bonatsou et al., 2015). Therefore, it contributes both to fruit debittering by phenolic compounds biodegradation, and reduction of the large quantities of olive wastewater produced when a lye treatment is applied (Arroyo-López et al., 2012c).

Among the other frequently isolated species, strains of *D. hansenii* and *S. cerevisiae* were reported, like many other less common yeasts species, to favor LAB development, both synthesizing vitamins, aminoacids and purins, or metabolizing complex carbon sources (Arroyo-López et al., 2012c). Moreover, *D. hansenii*, as *P. membranifaciens, C. boidinii*, and *W. anomalus*, showed strain-specific killer activity (toxic proteins or glycoproteins production) against spoilage yeasts (Psani and Kotzekidou, 2006; Arroyo-López et al., 2008, 2012c; Hernández et al., 2008). When selecting starter strains, this last one is an important feature to be investigated and searched foras well as the technological traits, because it allows to reduce salt content or chemical preservatives use, thus obtaining a more natural product with an improved shelf-life (Arroyo-López et al., 2012c).

### Table olives as carrier and source of probiotic LAB

Probiotics are defined as “Live microorganisms which, when administered in adequate amounts, confer a health benefit on the host” (FAO/WHO, 2001). Their claimed benefits are numerous. In fact, they are believed to be capable of: equilibrating the intestinal microbiota, thus reducing the risk of gastro-intestinal diseases; improving nutrients bioavailability; prevent or reduce allergies. Furthermore, they are presumed to have antimutagenic, anticarcinogenic (reduction of the risk of colon, liver, and breast cancers), hypocholesterolemic, antihypertensive, anti-osteoporosis, immunomodulatory effects (Behnsen et al., 2013; Kechagia et al., 2013; Papadimitriou et al., 2015). However, despite the increase of studies documenting these effects for strains isolated from different sources, benefits of foods containing probiotics are not taken for granted, but must be scientifically proven and certificated by the authorities in charge, before they can be claimed on the food labels. The European Parliament and Council of The European Union (2006), issued the EC Regulation N. 1924/2006 in order to protect consumers and facilitate their choice. At present, the only probiotic health claim approved for foods in EU, by the European Food Safety Authority (EFSA), is that related to live yogurt cultures, recognized to be able to “improve lactose digestion of the product in individuals who have difficulty digesting lactose” [EFSA, 2010]. In regard to USA, probiotics are not considered “health claims,” but “structure/function claims” (for instance “promote digestion” or “help maintain normal cholesterol levels”). Thus, while health claims need a pre-approval by US Food and Drug Administration (FDA), before the product is put on the market, the structure/function claims do not. However, manufacturers are responsible for their veracity and their label must show the following disclaimer: “This statement has not been evaluated by the Food and Drug Administration. This product is not intended to diagnose, treat, cure, or prevent any disease” (Hoffmann, 2013). At present, even in USA, no qualified health claim has been approved for any probiotic microorganism (Szajewska et al., 2016).

Nevertheless, consumers are more and more interested in functional products in general, including those that contain probiotic microorganisms.

In the last decades, various studies on probiotics conveyed by products of vegetal origin (i.e., fruit, fermented vegetables, cereals) were carried out (Granato et al., 2010; Peres et al., 2012; Abriouel et al., 2017; Huerta-Vera et al., 2017). Among vegetable products, table olives are considered a very interesting matrix to support the survival of both added selected commercial probiotic strains, and wild-type autochthonous ones that naturally colonize drupes and brines even during refrigerated shelf-life (Lavermicocca et al., 2005; Rodríguez-Gómez et al., 2014b, 2017; Argyri et al., 2015). Indeed, olives proved to support probiotics survival as or better than dairy products, and better than other vegetal substrates, maybe because of the nutrients release from the fruits, the presence of prebiotic substances, and their microstructure. In particular, the rough olive surface seems to protect bacteria from the acid environment, and favor the formation of mixed LAB and yeast communities biofilms, able to vehicle probiotics to human gastrointestinal (GI) tract (De Bellis et al., 2010; Ranadheera et al., 2010; Arroyo-López et al., 2012a; Blana et al., 2014; Rodríguez-Gómez et al., 2014a,b, 2017; Grounta et al., 2015).

Strains belonging to L. plantarum and L. pentosus, the most represented LAB starter species, among other less frequently isolated species (i.e., L. paracasei subsp. paracasei, L. casei, L. paraplantarum, Leuconostoc mesenteroides, and Ln. pseudomesenteroides), colonizing different cultivars of table olives (Conservolea, Halkidiki, Aloreña, Gordal, Manzanilla, Nocellara Etnea, and Bella di Cerignola), have been extensively tested for their *in vitro* probiotic characteristics (Bevilacqua et al., 2010; Argyri et al., 2013; Bautista-Gallego et al., 2013a; Botta et al., 2014; Pérez Montoro et al., 2016). Furthermore, potential probiotic strains have been studied also for their technological abilities, in order to develop multifunctional starters able to both control fermentation and grant probiotic characteristics to the product (Bevilacqua et al., 2010; De Bellis et al., 2010; Rodríguez-Gómez et al., 2013). In any case, when looking for probiotic strains, both functional characterization and safety assessment must be performed, following the FAO/WHO (2002) guidelines. First, strains intended to be added to food must have the QPS (in EU), or be GRAS (in USA), and, if intended to be administered to at-risk subjects, accomplish even more strict quality standards (Fijan, 2014; Sanders et al., 2016; Abriouel et al., 2017). The successive step should be checking their capacity to colonize the host. In fact, this is an indispensable prerequisite, which makes probiotics able to exert their beneficial effects (Kechagia et al., 2013; Papadimitriou et al., 2015). Therefore, isolates from naturally fermented table olives were tested for their ability to survive low pH values, resist bile acid, hydrolyse bile salts, adhere to mucus and/or human epithelial cells, and autoaggregate(Bevilacqua et al., 2010; Argyri et al., 2013; Bautista-Gallego et al., 2013a; Botta et al., 2014; Pérez Montoro et al., 2016). Moreover, haemolytic activity, antibiotic resistance and bacteriocin production as well as other important safety features were assessed. The latter characteristic is important to counteract pathogen development not only in human GIT, but also in food matrices (Pérez Montoro et al., 2016).

*L. plantarum, L. paracasei subsp. paracasei*, and, particularly, *L. pentosus* strains turned out to be the most promising probiotic species, sometimes showing even better performances than *L. rhamnosus* GG (LGG) and *L. casei* Shirota probiotic strains, used as control (Bevilacqua et al., 2010; Argyri et al., 2013; Bautista-Gallego et al., 2013a; Botta et al., 2014; Pérez Montoro et al., 2016).

However, *in vitro* tests positive results are not sufficient to demonstrate the actual *in vivo* efficacy of putative probiotic strains. Reduced risk of disease, improvement in illness symptoms, or well-being promotion, as a result of their use, must be proved through animal and clinical studies, in order to define a strain as probiotic (FAO/WHO, 2002). Unfortunately, regulations of probiotics is still quite vague. Often the same schemes/protocols used in drugs development are applied to probiotic foods, and this limiting approach, even though could be positive for consumers protection, may be responsible to restrain innovation and research on foods health benefits, preventing consumers to access to potential helpful information (van Loveren et al., 2012; Hill et al., 2014).

## Innovations in table olive processes and processing plants

### Salt reduction and replacement

As reported earlier in this article, current practices in Sevillan style processing are quite stable and widespread among producers, with the main technological applied innovation being the selection and use of starter cultures, as described above. On the other hand, in recent literature, a large amount of contributions on optimization of processing parameters of table olive dealt with NaCl reduction and partial substitution with other salts. Indeed, salt intake is a topic of concern, because its excessive consumption has been related to hypertension and cardiovascular diseases (World Health Organization (WHO), 2012), expecially in those countries where the daily intake of NaCl is substantially higher than the recommendations (U.S. Dept. of Agriculture U.S. Dept. of Health Human Services, 2010). Therefore, the formulation of low salt foods, including fermented vegetables, to improve consumers' health, is a challenge to be addressed by food scientists. Fadda et al. (2014) compared the impact of two different salt concentrations on the texture and antioxidant activity of natural table olives. The texture profile did not reveal any difference between treatments, while the antioxidant activity was preserved more in 7% salt brined table olives than in the 4%. Among NaCl replacers, calcium chloride, potassium chloride, and magnesium chloride are the most investigated, mainly because of the positive health effects of Ca, K, and Mg intake, especially associated with a low Na diet (Leiba et al., 2005), and their inclusion in the list of nutrients of food regulations (Bautista-Gallego et al., 2013b). The reduction of NaCl in fermentation brines of table olives is advisable, since it has positive effects on LAB growth, as reported in naturally black olives, (Tassou et al., 2002, 2007), depending also on NaCl concentration. Its partial substitution with CaCl_2_ has been reported to affect the olive texture (Tassou et al., 2007), and usually has a detrimental effect on *Enterobacteriaceae* (Bautista-Gallego et al., 2010) and Yeasts growth (Bautista-Gallego et al., 2015). A partial substitution of NaCl with other chlorine salts (CaCl_2_ and KCl) did not alter the fermentation profile of black olives, while substantially reducing the sodium content, improving the final product. On the other hand, other authors reported the effect of calcium addition on reducing the diffusion of soluble sugars from olive to brine, with a detrimental effect on acidity development and pH drop (Bautista-Gallego et al., 2011a; Rodríguez-Gómez et al., 2012). In Manzanilla olives, moderate (20–30 g/L) addition of CaCl_2_ can be advisably used in substitution of NaCl, in the presence of the same amount of KCl, being stimulating for LAB growth, positively affecting acidity development, while not stimulating the growth of *Enterobacteriaceae* (Bautista-Gallego et al., 2015). From a sensory point of view, the addition of other salts other than NaCl in the processing of natural green olives and black olives resulted in a reduced salt concentration product with acceptable sensory characteristics (Di Silva, 2000; Mulé et al., 2000), and no impact on saltiness perception when KCl, NaCl and CaCl_2_ are added in the same proportion to the brine (Marsilio et al., 2002). High levels of CaCl_2_, however, could lead to excessive hardness of tissues resulting in a negative impact on the overall sensory characteristics. In a study conducted on Conservolea natural black olives, only 4% KCl plus 4% NaCl, among the mixture tested, could finally produce olives with lower sodium content without affecting the “traditional” taste (Panagou et al., 2011). The use of ZnCl_2_ has also been proposed to fortify fermented vegetables and as an alternative preservative to potassium sorbate. Treatments have been shown to have a positive impact on texture, physico-chemical features, and sensory properties. Olive packaging with 0.75 g/L of ZnCl_2_ led to olives with higher final sugars concentration, due to selective inhibition of microflora, lower pH and higher titratable acidity of the brines, having a positive effect on sensory properties (Bautista-Gallego et al., 2011c). Bautista-Gallego et al. (2013c) obtained better scores for instrumental firmness and the lowest scores for the kinesthetic sensations, bitterness and saltiness, for olives treated with ZnCl_2_ (0.075% w/v) rather than those containing potassium sorbate (1.2% w/v).

### Optimization of processing factors and innovative processing plants

Natural green olives are processed through several months in order to reduce the bitterness at a palatable level and ensure chemical and microbiological stability. The main mechanism involved is the slow diffusion of bitter phenols from pulp to brine (Romero et al., 2004a,b; Arroyo-López et al., 2005). Many chemical-physical variables influence the natural process, namely the phenolic content in pulp, linked to the variety and degree of ripeness (Campus et al., 2015), salt concentration in brine (Fadda et al., 2014), acidification of brines, which can favor acid hydrolysis of oleuropein (Gikas et al., 2006; Servili et al., 2006), endogenous (Ramírez et al., 2016), and microbial hydrolases (Ciafardini et al., 1994; Servili et al., 2006), with β-glucosidase and esterase activity. Recently, alternative transformation protocols have been proposed in order to enhance the fermentation profile and accelerate the debittering of natural table olives. Ramírez et al. (2017) studied the effect of sodium chloride, acid concentration, and storage temperature on the enzymatic hydrolysis of oleuropein. Temperature optimum for endogenous β-glucosidases is cultivar dependent (Ramírez et al., 2014), with the pH-optimum around 5 (Romero-Segura et al., 2009; Kara et al., 2011; Ramírez et al., 2014), whileesterase activity decreases drastically at lower pH values. The cited authors proposed the following protocol: in the first stage (1 month of brining), a low concentration of NaCl (60 g/L) and acetic acid (2 g/L) together with a low storage temperature (10°C), which facilitates the action of endogenous enzymes (β-glucosidase and esterase), to favor the hydrolysis of the bitter phenol. In the second stage, concentrations and temperature of storage are raised (140 g/L NaCl, 16 g/L acetic acid and 40°C) to favor chemical hydrolysis of oleuropein, for a few months (Ramírez et al., 2017).

During processing, phenolic compounds present in the olive give rise to antimicrobial chemical species that influence the fermentation, affecting the growth of LAB and thus the chemical and microbiological stability (Medina et al., 2007). This argument is of concern only if olives are not treated with NaOH, since the treatments eliminate the phenolic compounds largely. In this regard, olives processed without the addiction of alkali exhibit different behaviors, depending on the variety. The addition of nutrients (MRS medium or Yeasts extract) during the fermentation of “difficult” varieties, such as Manzanilla, has been proposed to overcome the inhibitory activity of oleuropein-derived antimicrobial compounds (Medina et al., 2010).

In special conditions, such as processing plants located in cold zones, the use of starter cultures has been studied and physico-chemical variables optimized to drive the fermentation in such environments. Durán Quintana et al. (1999), using response surface methodology and *L. plantarum* starter cultures, reported that the most effective combination of factors were: initial pH of 5.0; 3%,w/v, NaCl; incubation at 12°C. Thus, normal fermentation processes can be obtained in cold regions when appropriate initial conditions and starter cultures are used.

In face of innovations proposed to drive the fermentation processes (Bevilacqua et al., 2015), scarce are the research contributions to the development of novel table olive processing plants. Usually, table olives are processed in closed static reactors with no strict control of processing parameters (temperature, salt concentration, acidity development), without any kind of automation in the control and monitoring of such parameters. An innovative processing plant, which allowed the real time control of main process parameters (pH, temperature, O_2_ and CO_2_ dissolved, salinity of brine) has been described recently (Campus et al., 2017). A starter driven process in the innovative plant, with continuous recirculation of brine, at controlled temperature, has been compared to static natural fermentation in plastic vats. The authors reported the evolution of relevant process parameters, chemico-physical determinations, instrumental texture, and microbiological analyses. In the innovative process, a more rapid acidification onset was observed, pH reaching lower values than the control. Inoculated autochthonous *L. pentosus* strains have multiplied rapidly, supplanting *Enterobacteriaceae* in short time. No differences were found between samples in the instrumental texture profile analysis parameters (TPA). Notably, olives processed in the innovative plant were debittered in 3 months, as depicted by HPLC determination of phenolic compounds and Descriptive Sensory Analyses results, while naturally fermented olives took over 180 days to reach an acceptable level of bitter taste. Slight differences were found in few other sensory descriptors.

## Innovative post processing techniques

After the fermentation, the table olives are packed in glass or plastic containers, tins, and plastic (single- or multi-laminated) or aluminum pouches, to improve their economic value and expand markets. These packaging materials are filled with brine, which may contain additives for improving the microbial quality, and protecting sensorial features (Panagou, 2004; Degirmencioglu, 2011a; Degirmencioglu et al., 2014). In some cases, modified atmosphere packaging (MAP), vacuum packaging (VP), active packaging (AP), and packaging by edible coating and film (ECP) (Ščetar et al., 2010), can alternatively be used.

### Modified atmosphere packaging (MAP) and vacuum packaging (VP)

MAP is one of the most important food preservation methods. It consists in creating an atmosphere composed by CO_2_, O_2_, and /or N_2_ in a pack, regulating the ripening process, extending shelf life, controlling or reducing microbial spoilage during storage and distribution (Valdés et al., 2014). Vacuum packaging is an efficient method in which the food is packed with low oxygen permeability plastic film, then air is evacuated, and the package sealed. However, the gaseous atmosphere of the vacuum package can change due to microbial metabolism and chemical reactions within the packed food; the oxygen from the external environment can permeate into the packaging; therefore, over time, the atmosphere in the package may be different from the original atmosphere (Degirmencioglu et al., 2014). The use of MAP and VP for extending shelf life of table olives, at ambient or low temperature, has been well noted and proven in several studies (Panagou et al., 2002; Panagou, 2006; Degirmencioglu, 2011a; Degirmencioglu et al., 2011b, 2014; Mantzouridou and Tsimidou, 2011; Doulgeraki et al., 2012; Argyri et al., 2015). As reported by these researchers, dipping the olives in anti-microbial solutions before packaging and storing them under pressure in a CO_2_ atmosphere, at 4°C, controlled microbial activity effectively and minimized the production of mycotoxins, retaining the quality characteristics of the packed products.

### Edible coating packaging (ECP)

ECP have been used for preserving and extending shelf life of foods. Semi-permeable films or coatings are formed on the surface of foods, to control moisture loss, suppress respiration, and carry out other functions. These films or coating are made by dipping in or spraying on foods a proper solution which, after time, forms a continuous layer in contact with the food surface. In producing edible coatings, emulsifiers and plasticizers are often used for improving coating performances. Edible coatings and films may act as carriers of antioxidants, antimicrobials, flavoring and coloring compounds, which are added to enhance food stability, safety and quality (Ščetar et al., 2010; Moutsatsou et al., 2011; Khalifa et al., 2016). Also, several antioxidant or antimicrobial agents can be coated, incorporated, immobilized, or surface modified into edible coatings, such as organic acids, essential oils, fatty acid esters, polypeptides, phenolic compounds, nitrites, and sulfites (Ščetar et al., 2010; Carpiné et al., 2015; Martillanes et al., 2017).

Nowadays, the modern trend is to use natural polymers, such as chitosan, incorporated with natural additives, such as vanillin, olive leaf extract, cinnamdehyde, eugenol, carvacrol or cellulose derivates, such as carboxymethyl cellulose and methyl cellulose, as food coating material. Chitosan and hydroxy-propyl-methyl-cellulose (HPMC) were tested in combination with MAP (100% N_2_, 80% CO_2_ - 20% Air) by Moutsatsou et al. (2011), with the aim to propose a new type of preservation and packaging of table olives. The coating was successful in reducing the weight loss, maintenaning color and firmness and extending shelf life of the olives, while chitosan proved to be more efficient than HPMC as olives coating, when they were packaged in 100% air. Ramírez et al. (2015a) studied the combined effect of an edible coating (commercial coatings employed to prevent citrus post harvest deseases) and a nitrogen atmosphere to avoid the occurrence of brown spotting of Manzanilla olives mechanically harvested and processed as Spanish-style green olives. The percentage of olives free of any brown spots ranged between 35–50, 10–25, and 50–65% for fruit directly processed, stored under nitrogen, or coated and stored under nitrogen, respectively. Therefore, the postharvest storage of coated olives under nitrogen resulted to be a good method to prevent bruise damage in mechanically harvested fruit.

### Non-thermal processes

Decontamination and shelf life extension of foods are closely related to microbial quality and biochemical and enzymatic reactions. The non-thermal technologies, such as high hydrostatic pressure (HHP), ultraviolet (UV), ultrasound, osmotic dehydration, or irradiation, are used for controlling the growth of microorganisms and the sensory, physical and chemical parameters, during food processing and storage (Tokuşoglu et al., 2010; Delgado-Adamez et al., 2013; Manzocco and Nicoli, 2015).

Contamination of olives may be due to inadequate hygienic conditions (raw material, water, additives, fermentation vehicle, personel etc.) during harvest, pre-treatment, fermentation/processing and storage stages (Argyri et al., 2014b). Most fermented or processed olives are distributed to market “in bulk” and they could be exposed to high risk of contamination from the environment (Panagou et al., 2013; Argyri et al., 2014b). The final product may be marketed or exported safely in packaging materials (Degirmencioglu, 2011a; Panagou et al., 2013) by using a thermal pasteurization or physical packaging methods, for extending the shelf life (Kadam et al., 2012).

Among the non-thermal technologies, high hydrostatic pressure (HHP) is applied to liquid and solid foods, in the range of 50–1,000 MPa (Delgado-Adamez et al., 2013; Argyri et al., 2014b). Generally, moderate pressure (up to 200–300 MPa) decreases the rate of reproduction and growth of microorganisms, whereas higher pressure (300–700 MPa) inactivates microbial activity (Tokuşoglu et al., 2010), largely in dependence of the target microorganism. More recently, HHP has been used in a wide variety of applications; however, there are only a limited number of studies on the application of HHP to fermented vegetable foods, such as table olives (Tokuşoglu et al., 2010; Pradas et al., 2012; Abriouel et al., 2014; Argyri et al., 2014b).

Olives have a high content of functional micronutrients, fatty acids, bioactive phytochemicals (polyphenols, tocopherols, and phytosterols), having antioxidant, anti-inflammatory, antimicrobial properties, which have well-known positive effects on human health (Tokuşoglu et al., 2010; Delgado-Adamez et al., 2013). On the other side, table olives act as a suitable substrate for the production of mycotoxins (especially citrinin) produced by filamentous fungi *Penicillium, Aspergillus*, and *Monascus* (Tokuşoglu et al., 2010). Nevertheless, thermal processing of table olives induces some quality deterioration, resulting in softening of olive tissue, loss of green color or change to brown, and development of cooking taste (Dimou et al., 2013; Abriouel et al., 2014). For this purpose, optimization of the HHP conditions is important for bioactive compounds stability and quality of table olives, as an alternative to thermal processing (Argyri et al., 2014b).

Different levels of HHP (250–600 MPa for 5–30 min) showed to be more effective than thermal pasteurization (80°C for 20 min) in reducing the yeast and mold populations, the mycotoxin level (citrinin), and extending the shelf life of table olives and derived products (Tokuşoglu et al., 2010; Sánchez et al., 2012; Delgado-Adamez et al., 2013; Abriouel et al., 2014; Argyri et al., 2014b). Notably, the total phenolic and hydroxytyrosol levels were increased by factors of 2.1–2.5 and 0.8–2.0, respectively, while oleuropein decreased after HHP (Tokuşoglu et al., 2010). Similar results were also reported for Cornezuelo olives treated by HHP (400 MPa for 5 and 10 min and 600 MPa for 5 and 10 min). Olives treated by HHP had higher stability in terms of pH and free acidity values, and the HHP treatment (400 MPa for 5 min) can be used to prevent the formation of gas in the packed olives and to improve the sensory characteristics of Cornezuelo dressed olives (Pradas et al., 2012).

The color makes a key contribution to the marketability of table olives, and the vivid green color is an essential characteristic of the product, especially in Spanish-style processing (Sánchez-Gómez et al., 2013; Argyri et al., 2014b); it must be noted that HHP treatments caused a moderate degradation of the color of the processed olives (Pradas et al., 2012; Delgado-Adamez et al., 2013; Abriouel et al., 2014; Argyri et al., 2014b). For preserving and improving the color of HHP-treated olives, the addition of ascorbic acid (15 g/L) or purging with gaseous nitrogen has been suggested (Arroyo-López et al., 2008).

### Biopreservation

Biopreservation is the use of microorganisms and their metabolites (organic acids, ethanol, carbon dioxide, antifungal compunds, bacteriocins, etc.) with the aim of extending the shelf life and improving the safety of foods (Settanni and Corsetti, 2008; Di Cagno et al., 2013). In particular, biopreservation by use of LAB cultures and their metabolites, including bacteriocins, has gained interest beacause of their antibacterial (Chen and Hoover, 2003; Bhattacharyya and Bhattacharjee, 2007) and antifungal activity (Schnürer and Magnusson, 2005). LAB with biopreservation activity should conveniently originate from the same type of food material as they are intended for use in (Vescovo et al., 1995, 1996). LAB strains involved in the fermentation of table olives were found to produce bacteriocins, and to being active *in vitro* against other LAB strains and against various genera of bacteria, including spoilage and pathogenic ones, such as Propionibacterium, Clostridium (Jiménez-Díaz et al., 1993; Delgado et al., 2005; Ruiz-Barba et al., 2010), and Helicobacter pylori (Brito et al., 2012). Studies has proven that some strains isolated from brined table olives can adhere to porcine jejune epithelial cells (Bevilacqua et al., 2010), and survive low pH and bile concentrations, as previously stated in section Table olives as carrier and source of Probiotic LAB, exherting probiotic activity (see section Table olives as carrier and source of Probiotic LAB). The ideal biopreserving bacteria should successfully lead successfully the fermentation, supplant spoilage and pathogenic microrganisms, and be active (or its antimicrobial metabolites) also during shelf life. Among Yeasts, the species Wickerhamomyces anomalus and Pichia membranifaciens have inhibitory activity against a considerable number of microorganisms (Santos et al., 2000; Passoth et al., 2011), as extensively reviewed in section Yeasts with positive technological traits.

## Conclusions

The improvement of processing technologies is an essential step toward better, safer and more profitable table olive productions. In this review, conventional and advanced pre-processing operations, transformation and post packaging interventions have been reviewed and discussed. For sanification purposes, alternative to chlorine agents, alone or combined with other treatments, have been experimented and, in suitable conditions, proved to be effective in reducing the starting contamination of microbes and filth, although more data are needed to evaluate the suitability of innovative techniques such as ionizing radiations, unltrasounds and electrolyzed water solutions. Chlorine oxyde and ozone have been also employed to effectively reduce pesticide in table olive under law level. Starter cultures are the main applied biotechnological innovation in table olive processing. LAB and yeasts are the main microbial groups studied, and several strains have been selected and characterized for their technological traits. LAB strains with positive traits are capable to drive the fermentation process, supplanting spoilage microflora and favoring the onset of physical-chemical conditions that ensure microbial stability. Yeasts can exhert positive or negative effects, depending on the species and strains involved. Authoctonous starters have advantages over single strain starters, in therms of adaptability and characterization of typical table olive preparations. Researchers have paid attention in the enhancement of healthiness of table olive, considered as a potential media to vehicle probiotics. Moreover, in recent literature, many contributions on processing parameters optimization dealt with NaCl reduction. Due to its implication in human health, partial sodium salt replacement with other salts have been showed to be possible and advisable at the same time. Alternative processing protocols have been described, in order to shorten time or obtain olives in disfavourable climate conditions. The contributions dealing with innovative processing plants are scarce, although incouraging results have been published recently. Among packaging interventions, MAP is the most promising and industrializable technique to prolong shelf life. Thermal post packaging treatment, such as pasteurization, are effective in reducing microbial contamination, but have detrimental effects on canned products. HPP has been proposed as an effective non thermal stabilization technique, which ensures microbial and enzymatic stability, minimizing the impact over sensory and nutritional features. As a concluding remark, bioremediation gives interesting perpsectives of a multifintional use of starter cultures, not only to drive the fermentation process but also to guarantee table olive preservability over time.

## Author contributions

MC conceived the idea for the article, prepared and edited sections Table Olive Processing Technologies Up to Date and Innovations in Table Olive Processes and Processing Plants. ND prepared and edited sections Pre-processing of Olives and Innovative Post-processing Techniques. RC prepared and edited section Biotechnological Innovations and the tables. All authors reviewed the manuscript and approved it for publication.

### Conflict of interest statement

The authors declare that the research was conducted in the absence of any commercial or financial relationships that could be construed as a potential conflict of interest.
